# A Two-Dimensional Eight-Node Quadrilateral Inverse Element for Shape Sensing and Structural Health Monitoring

**DOI:** 10.3390/s23249809

**Published:** 2023-12-14

**Authors:** Mingyang Li, Erkan Oterkus, Selda Oterkus

**Affiliations:** 1 Ocean College, Jiangsu University of Science and Technology, Zhenjiang 212100, China; 202200000174@just.edu.cn; 2 Department of Naval Architecture, Ocean and Marine Engineering, University of Strathclyde, Glasgow G4 0LZ, UK; selda.oterkus@strath.ac.uk

**Keywords:** iFEM, shape sensing, structural health monitoring, quadrilateral, two-dimensional

## Abstract

The inverse finite element method (iFEM) is a powerful tool for shape sensing and structural health monitoring and has several advantages with respect to some other existing approaches. In this study, a two-dimensional eight-node quadrilateral inverse finite element formulation is presented. The element is suitable for thin structures under in-plane loading conditions. To validate the accuracy and demonstrate the capability of the inverse element, four different numerical cases are considered for different loading and boundary conditions. iFEM analysis results are compared with regular finite element analysis results as the reference solution and very good agreement is observed between the two solutions, demonstrating the capability of the iFEM approach.

## 1. Introduction

Shape sensing and structural health monitoring (SHM) are effective approaches to ensure the safety of structures by utilising sensor systems, collecting sensor data, processing the data, and finally making decisions. There are various shape sensing approaches available in the literature. The Amongst Model Method [1] can make predictions without material information and it is suitable for both beam- and plate-type structures. On the other hand, Ko’s Displacement Theory [2] is suitable for beam-type structures. Another promising approach is the inverse finite element method (iFEM) [3]. iFEM is based on discretisation of the solution domain by using suitable inverse elements, such as the beam, plate, shell, or solid, and utilising collected strain data from sensors located on the structure. iFEM is a robust approach and can be used for real-time monitoring for complex structures. Moreover, there is no need to measure loading during the monitoring process.

There has been a significant progress throughout the years in iFEM methodology. Various different types of inverse elements have been developed for different types of structures. Tessler and Spangler [4] developed a three-node inverse shell element (iMIN3) which is based on the variation in in-plane displacements and bending rotations linearly and transverse displacements quadratically along with in-plane coordinates. The capability of this element has also been extended for large deformations [5]. An inverse beam element based on Timoshenko beam formulation was developed by Gherlone et al. [6]. A four-node shell element with drilling degree-of-freedom (iQS4) was introduced by Kefal et al. [7] and has been successfully utilised for the monitoring of various marine structures [8,9]. An eight-node curved shell element (iCS8) based on the first-order deformation theory was developed by Kefal [10]. A novel inverse solid element formulation was presented by de Mooij et al. [11] and various benchmark problems were considered. iFEM has also been applied for composite materials. Plate and shell elements suitable for composite and sandwich structures were developed by Cerracchio et al. [12] and Kefal et al. [13]. iFEM has also been used for damage prediction in structures [14]. Kefal and Oterkus [15] introduced isogeometric iFEM analysis to reduce the number of required sensors for iFEM analysis, which was further explored in some other studies [16,17].

In this study, a two-dimensional eight-node quadrilateral inverse finite element formulation is presented. The element is suitable for thin structures under in-plane loading conditions. The element has a computational advantage with respect to a shell element because each node has two degrees of freedom with respect to six degrees of freedom for a shell element if the dominant loading and deformations occur on a particular plane. To validate the accuracy and demonstrate the capability of the inverse element, four different numerical cases are considered by considering different loading and boundary conditions. iFEM analysis results are compared with regular finite element analysis results as the reference solution.

## 2. Materials and Methods

**Figure 1:**
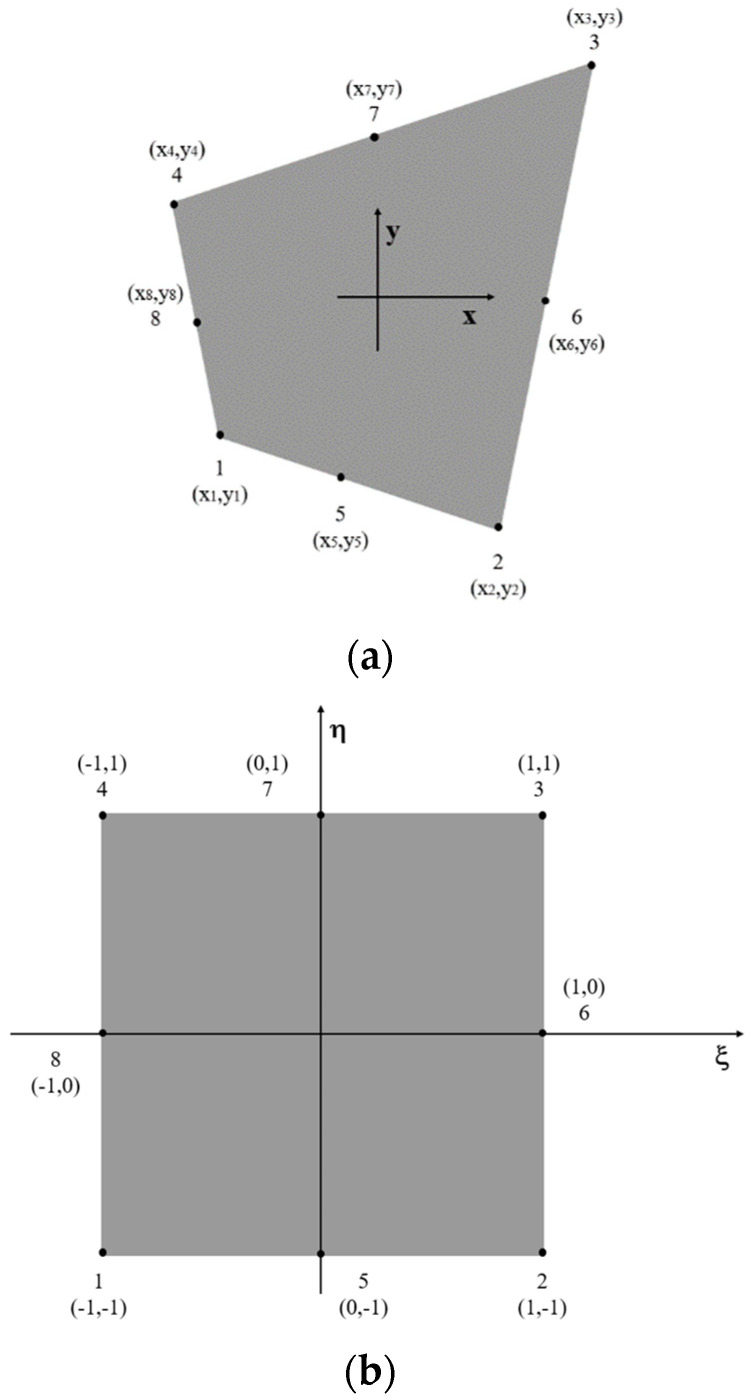
(**a**) Two-dimensional eight-node quadrilateral inverse element, (**b**) the master element in (ξ,η) space.

In this section, the details of the formulation for a two-dimensional eight-node quadrilateral inverse element, named as iQP8, are provided. As shown in Figure 1a, each node has two degrees of freedom, u and v, corresponding to in-plane displacements in the *x* and *y* directions, respectively. The master element has a square shape and is defined in the natural coordinate system (ξ,η), as depicted in Figure 1b.

The location of any point on the iQP8 element can be expressed in terms of the location of nodes in the (x,y) coordinate system, $\left(x_{i},y_{i}\right)$, and bilinear isoparameteric shape functions, $N_{i}(\xi ,\eta )$, as

(1a) $$x(x,y)=\sum_{i=1}^{8}N_{i}x_{i}$$

(1b) $$y(x,y)=\sum_{i=1}^{8}N_{i}y_{i}$$

The bilinear isoparameteric shape functions, $N_{i}(\xi ,\eta )$, are defined as

(2a) $$N_{1}=\frac{\left(1-\xi )(1-\eta )(-1-\xi -\eta \right)}{4}$$

(2b) $$N_{2}=\frac{\left(1+\xi )(1-\eta )(-1+\xi -\eta \right)}{4}$$

(2c) $$N_{3}=\frac{\left(1+\xi )(1+\eta )(-1+\xi +\eta \right)}{4}$$

(2d) $$N_{4}=\frac{\left(1-\xi )(1+\eta )(-1-\xi -\eta \right)}{4}$$

(2e) $$N_{5}=\frac{\left(1-\xi )(1+\xi )(1-\eta \right)}{2}$$

(2f) $$N_{6}=\frac{\left(1+\xi )(1+\eta )(1-\eta \right)}{2}$$

(2g) $$N_{7}=\frac{\left(1-\xi )(1+\xi )(1+\eta \right)}{2}$$

(2h) $$N_{8}=\frac{\left(1-\xi )(1+\eta )(1-\eta \right)}{2}$$

Similarly, by using the same shape functions, the in-plane displacements, u and v, at any point (x,y) can be written in terms of nodal displacements, $u_{i}$ and $v_{i}$, as

(3a) $$u(x,y)=\sum_{i=1}^{8}N_{i}u_{i}$$

(3b) $$v(x,y)=\sum_{i=1}^{8}N_{i}v_{i}$$

Strain components can be obtained by using the relationships between the strain and displacement components. For a plane element, only three components of in-plane strains can be expressed as

(4a) $$\varepsilon_{xx}=\frac{\partial u}{\partial x}$$

(4b) $$\varepsilon_{yy}=\frac{\partial v}{\partial y}$$

(4c) $$\gamma_{xy}=\frac{\partial u}{\partial y}+\frac{\partial v}{\partial x}$$

By utilising the displacement expressions given in Equation (3a,b) and strain definitions given in Equation (4a–c), the analytical elemental strains can be expressed by the shape functions and nodal displacements as

(5) $$e(u^{e})=\left\{\begin{array}{c} \varepsilon_{xx}\\ \varepsilon_{yy}\\ \gamma_{xy} \end{array}\right\}=B^{m}u^{e}$$

where $u^{e}$ are the nodal displacements, $u^{e}={\left[\begin{array}{cccccccccccccccc} u_{1} & v_{1} & u_{2} & v_{2} & u_{3} & v_{3} & u_{4} & v_{4} & u_{5} & v_{5} & u_{6} & v_{6} & u_{7} & v_{7} & u_{8} & v_{8} \end{array}\right]}^{T}$. $B^{m}$ is the matrix formed by the shape functions of each node as $B^{m}={\left[\begin{array}{cccccccc} B_{1}^{m} & B_{2}^{m} & B_{3}^{m} & B_{4}^{m} & B_{5}^{m} & B_{6}^{m} & B_{7}^{m} & B_{8}^{m} \end{array}\right]}^{T}$. Every single $B_{i}^{m}$ matrix can be defined as

(6) $$B_{i}^{m}=\left[\begin{array}{l} \begin{array}{cc} \frac{\partial N_{i}}{\partial x} & 0 \end{array}\\ \begin{array}{cc} 0 & \frac{\partial N_{i}}{\partial y} \end{array}\\ \begin{array}{cc} \frac{\partial N_{i}}{\partial y} & \frac{\partial N_{i}}{\partial x} \end{array} \end{array}\right]$$

The iFEM solution can be obtained by minimizing a weighted least-squares functional with respect to nodal degrees of freedom for the entire solution domain. For each inverse element, the weighted least-squares function can be written as

(7) $$\phi^{e}(u^{e})=w_{e}{\left\|e(u^{e})-e^{inputs}\right\|}^{2}$$

where $w_{e}$ is the weighting coefficient and the $e^{inputs}$ vector contains the measured strain input data. If the experimental strains for the element are available, then $w_{e}=1$. However, if the data are missing, a small value like $10^{-3}$ or $10^{-4}$ would be preferred. The squared norm in Equation (7) can be expressed as

(8) $${\left\|e(u^{e})-e^{inputs}\right\|}^{2}=\frac{1}{n}\int \int_{A^{e}}\sum_{i=1}^{n}{\left(e{\left(u^{e}\right)}_{i}-e_{i}^{inputs}\right)}^{2}dxdy$$

where $A_{e}$ is the area of the element and n is the number of locations for measured strains in an element. Minimizing the differences between the analytical strains and experimental strains for each element yields

(9a) $$\frac{\partial \phi^{e}(u^{e})}{\partial u^{e}}{=k}^{e}u^{e}-f^{e}=0$$

or

(9b) $$k^{e}u^{e}=f^{e}$$

where $k^{e}$ is the left-hand-side matrix and $f^{e}$ is the right-hand-side vector generated by the strain inputs which can be respectively expressed as

(10a) $$k^{e}=\int \int_{A^{e}}w_{e}{\left(B^{m}\right)}^{T}B^{m}dxdy$$

(10b) $$f^{e}=\frac{1}{n}\int \int_{A^{e}}\sum_{i=1}^{n}\left(w_{e}{\left(B^{m}\right)}^{T}e_{i}^{inputs}\right)dxdy$$

Next, the global equation system can be written based on the element contributions given in Equation (10a,b) as

(11) KU=F

where

(12a) $$K=\sum_{e=1}^{Nel}k^{e}$$

(12b) $$F=\sum_{e=1}^{Nel}f^{e}$$

(12c) $$U=\sum_{e=1}^{Nel}u^{e}$$

and Nel is the number of inverse elements. After applying displacement boundary conditions, the global equation system will take a reduced form as

(13) $$K_{R}U_{R}=F_{R}$$

where $K_{R}$, $U_{R}$, and $F_{R}$ are the reduced global left-hand-side matrix, displacement vector, and right-hand-side vector. Nodal displacements can be obtained by solving the equation system given in Equation (13). Once the nodal displacements are known, strains and stresses can be obtained similar to the approach used in regular finite element analysis (FEA).

**Figure 2:**
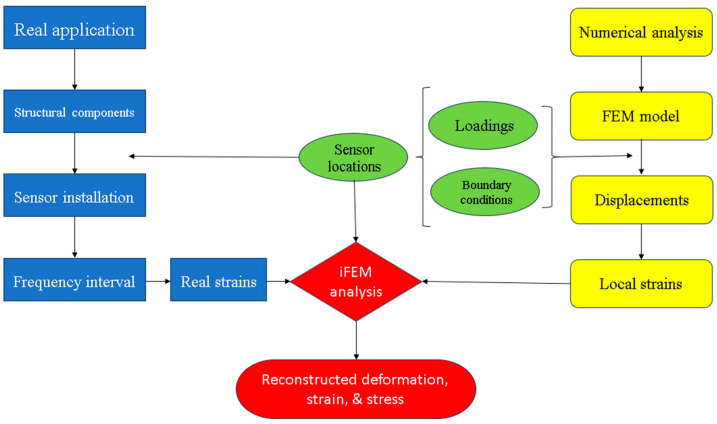
Flowchart describing the iFEM analysis process.

An important consideration for iFEM analysis is to determine the suitable sensor locations. To achieve this, synthetic sensor data can be generated by using regular FEA. FEA results can also be used as a reference solution. After determining the optimum sensor locations, sensors can be installed at those locations and data can be collected for iFEM analysis. Details of this process can be seen in Figure 2.

## 3. Results

We considered four different cases to validate the iQP8 inverse plane element formulation and demonstrate its capability, which are listed in Table 1, for different loading and boundary conditions. Furthermore, different mesh configurations together with reduced sensor conditions for fine mesh cases were also taken into consideration.

The influence of the mesh size will be explored in Case 1 and Case 2. Case 3 and Case 4 are introduced for further verifying the accuracy of the iQP8 inverse plane element and sensor selection. The results of displacements in two directions are mainly selected for comparison. For complex structure and loading conditions, von Mises stress is a useful parameter especially for potential failure of the structure. For the general plane stress condition, the von Mises stress can be calculated as

(14) $$\sigma_{vm}=\sqrt{\sigma_{xx}^{2}-\sigma_{xx}\sigma_{yy}+\sigma_{yy}^{2}+3\sigma_{xy}^{2}}$$

where $\sigma_{xx}$, $\sigma_{yy}$, and $\sigma_{xy}$ are in-plane stress components.

### 3.1. Case 1: Square Plate under Tension Loading

**Figure 3:**
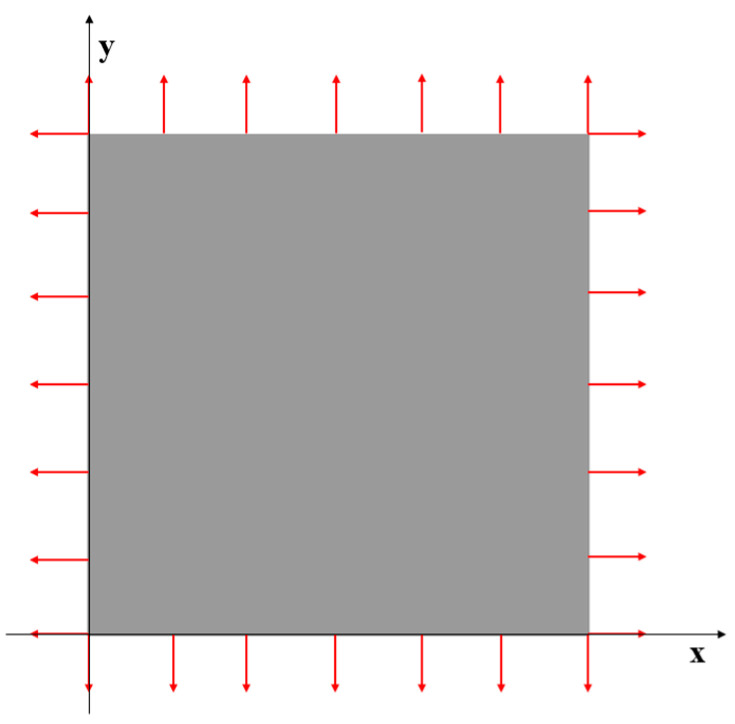
The loading of Case 1.

**Figure 4:**
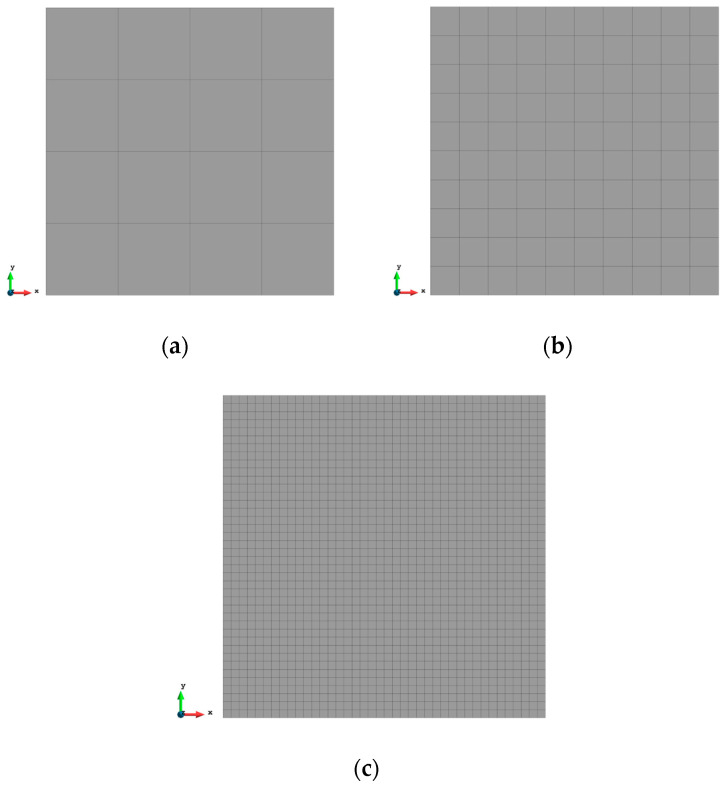
Three different meshes of Case 1, (**a**) 16 elements, (**b**) 100 elements, and (**c**) 1600 elements.

The first case is a square plate (2×2 m) under tension loading as shown in Figure 3. A force of 1000 MN is evenly distributed to the nodes of each edge of the plate. The plate is meshed with three different numbers of elements which are 16, 100, and 1600 (see Figure 4). The results of the three mesh cases are listed from Table 2, Table 3 and Table 4.

**Figure 5:**
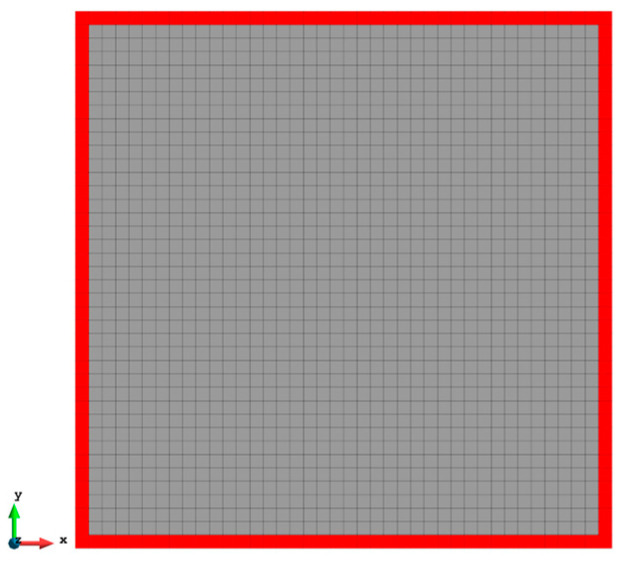
The reduced sensor locations of Case 1 with 1600 elements (iFEM-r).

For the mesh configuration with 16 elements, as can be seen from Table 2, iFEM displacement results have 4.279% error with respect to FEM results. With the increase in the number of elements, the percentages of difference of displacements are reduced dramatically to 2.193% for 100 elements. For 1600 elements, the reduced sensor condition is applied to the fine mesh case. As shown in Figure 5, only sensors along the edges of the plate are selected, which finally gives the number of sensors as 156. With the strain inputs provided by these 156 sensors, even if the strain data for the remaining elements are missing, the iQP8 element can still provide accurate results and the percentages of the error are just slightly raised from 0.616% to 0.784%.

**Figure 6:**
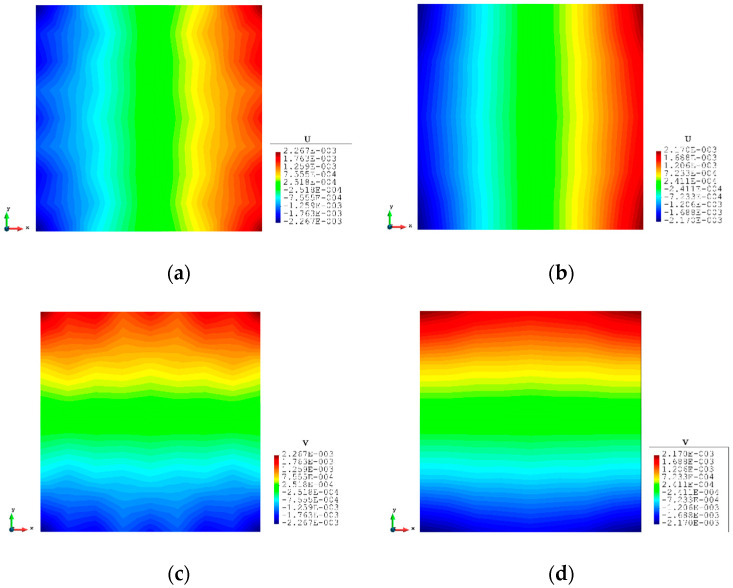
The plots of displacements of Case 1 with 16 elements: (**a**) x displacements of FEM, (**b**) x displacements of iFEM, (**c**) y displacements of FEM, (**d**) y displacements of iFEM.

**Figure 7:**
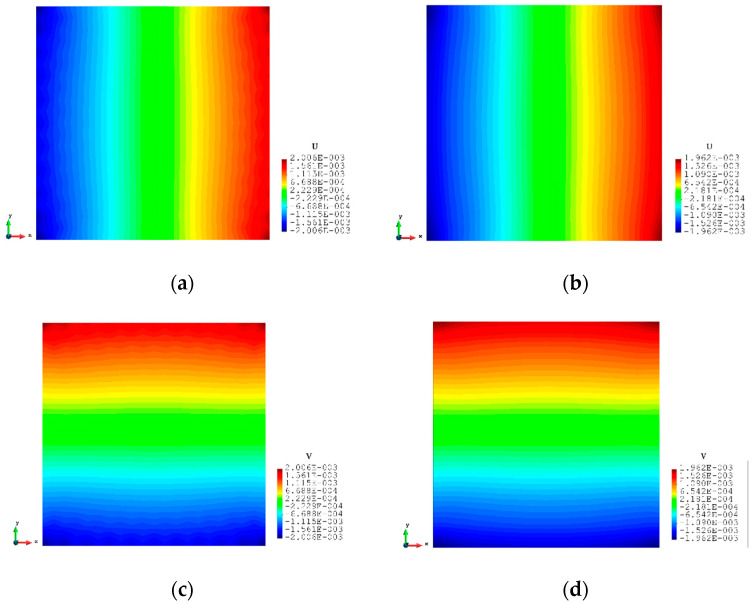
The plots of displacements of Case 1 with 100 elements: (**a**) x displacements of FEM, (**b**) x displacements of iFEM, (**c**) y displacements of FEM, (**d**) y displacements of iFEM.

**Figure 8:**
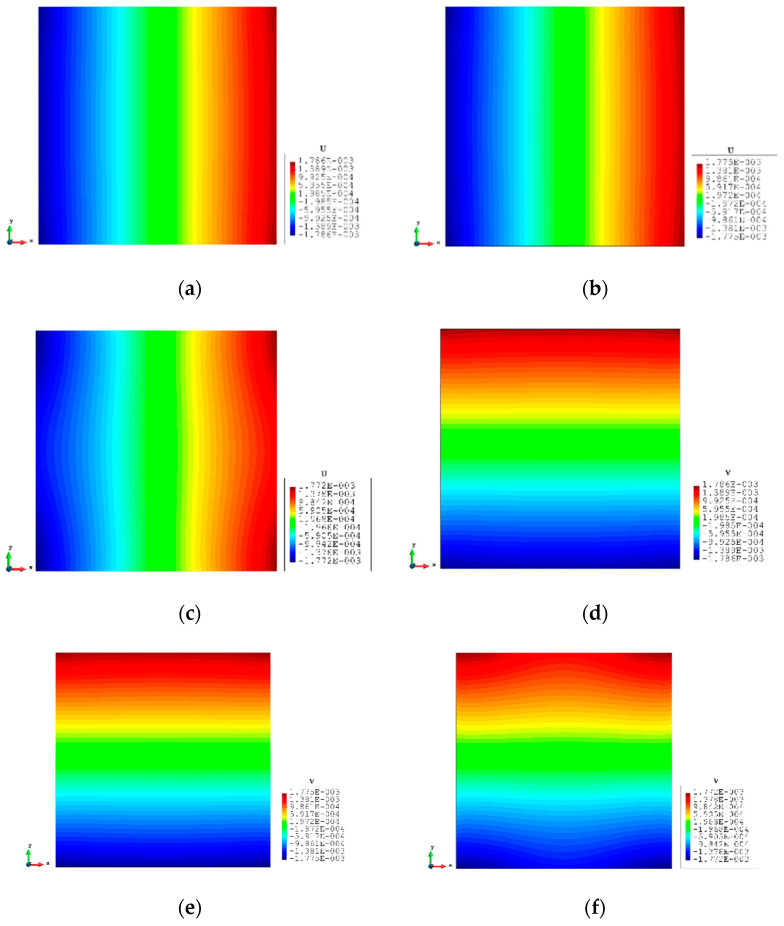
The plots of displacements of Case 1 with 1600 elements: (**a**) x displacements of FEM, (**b**) x displacements of iFEM, (**c**) x displacements of iFEM-r, (**d**) y displacements of FEM, (**e**) y displacements of iFEM, (**f**) y displacements of iFEM-r.

The contour plots of the displacements are also shown from Figure 6, Figure 7 and Figure 8 to further illustrate the results. It can be seen that the displacements are symmetrical along the central axis of the plate and the maximum/minimum values appear on the corners of the plate. These typical features can be captured by the inverse analysis, and they are not affected by the mesh and match well with the FEM plots including the reduced sensor condition (iFEM-r).

### 3.2. Case 2: Rectangular Plate under Tension Loading

**Figure 9:**
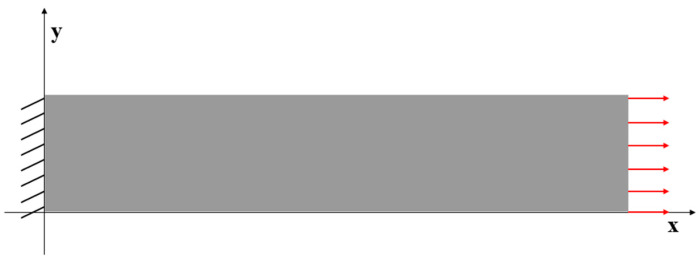
The loading and displacement boundary conditions of Case 2.

**Figure 10:**
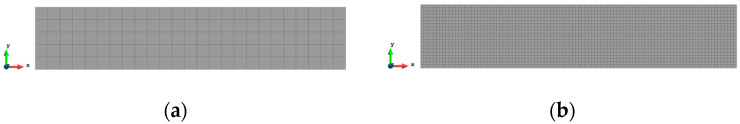
Two different meshes of Case 2: (**a**) 125 elements, (**b**) 2000 elements.

For Case 2, a rectangular plate, with 5 m length and 1 m height, is fully constrained on the left edge and the same tension loading as in Case 1 is applied to the right edge (see Figure 9). Similarly, the plate is meshed with both coarse mesh (125 elements) and fine mesh (2000 elements) (see Figure 10). Table 5 and Table 6 present the results for Case 2. If the mesh is quite coarse, the estimation of the y displacements is not as good as that of the x direction. The error of the v displacements is about 26.439% because the displacements in the y direction are much smaller than the displacements in the x direction (over 15 times). For the major displacement, u, reasonable results are obtained with an error of 2.245%, which means that the results would be acceptable. If the plate is meshed with 2000 elements, the displacements, especially in the y direction, are improved. The percentage of the differences is drastically dropped to around or less than 10%, which shows that inverse finite element results are approaching the reference FEM results.

**Figure 11:**
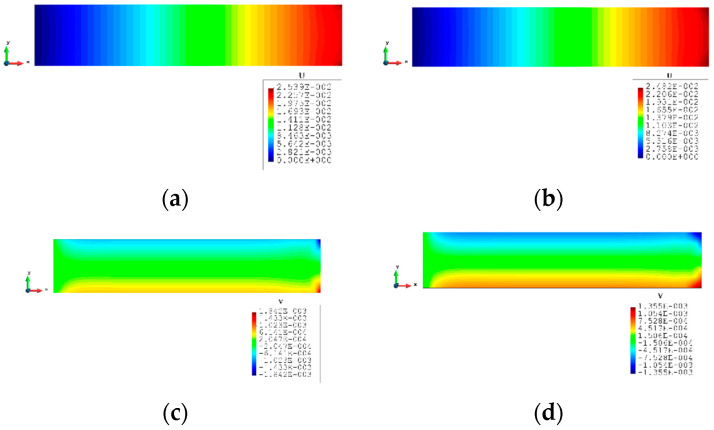
The plots of displacements of Case 2 with 125 elements: (**a**) x displacements of FEM, (**b**) x displacements of iFEM, (**c**) y displacements of FEM, (**d**) y displacements of iFEM.

**Figure 12:**
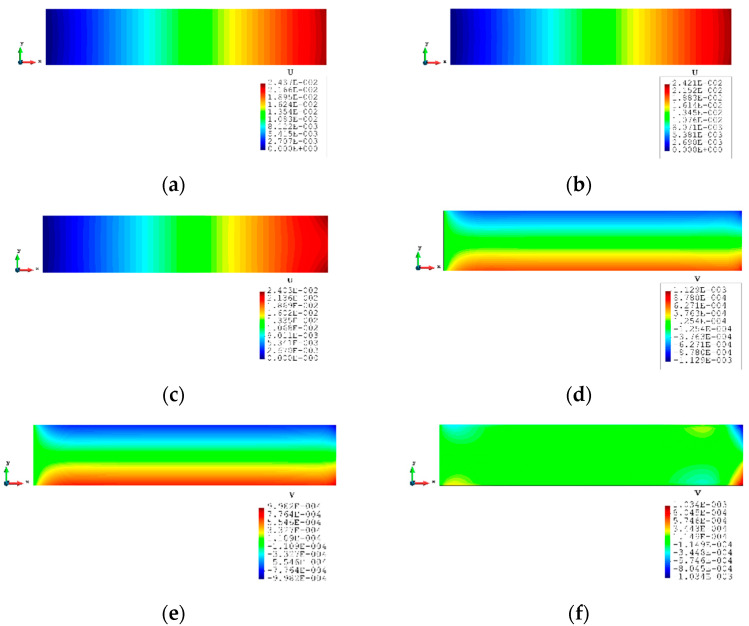
The plots of displacements of Case 2 with 2000 elements: (**a**) x displacements of FEM, (**b**) x displacements of iFEM, (**c**) x displacements of iFEM-r, (**d**) y displacements of FEM, (**e**) y displacements of iFEM, (**f**) y displacements of iFEM-r.

**Figure 13:**
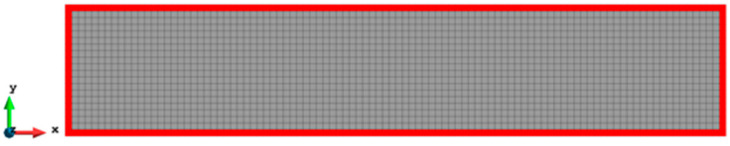
The sensor locations of Case 2 with 2000 elements.

The contour plots of Case 2 are given in Figure 11 and Figure 12. There is no doubt that, for the full sensor condition, the differences in the plots between the inverse analysis and FEM reference are indistinguishable. For the plots of the reduced sensor condition, the sensors are kept along the edge leading to a total number of 236 sensors (see Figure 13). Because of the sensor reduction, some features along the edge are not captured clearly. But the main features, i.e., the locations of the large deformations, are obviously captured.

### 3.3. Case 3: Rectangular Plate Subjected to a Nodal Force

**Figure 14:**
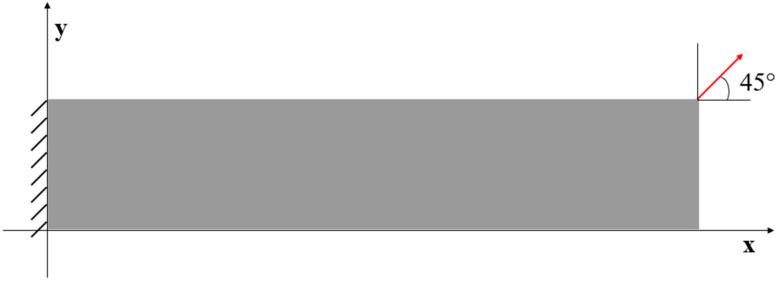
The loading and displacement boundary conditions of Case 3.

**Figure 15:**
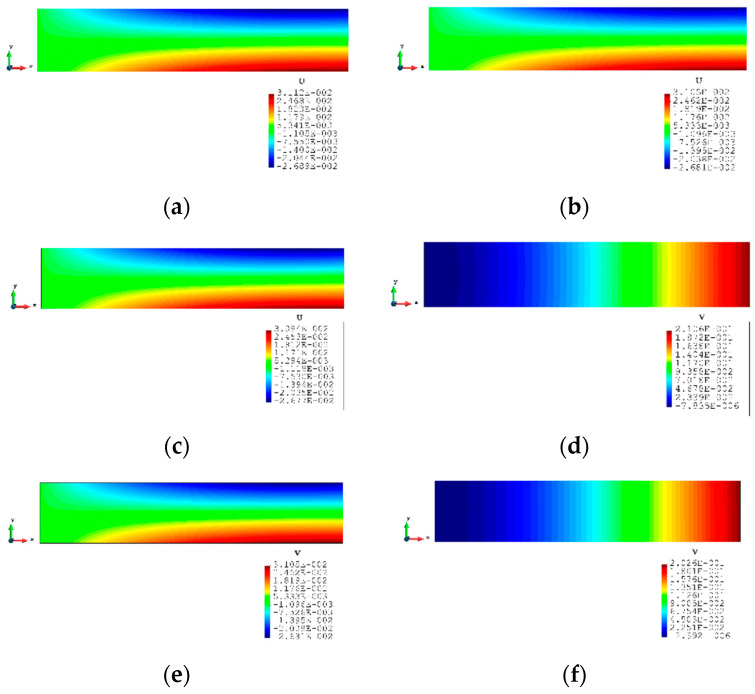
The plots of displacements of Case 3 with 2000 elements: (**a**) x displacements of FEM, (**b**) x displacements of iFEM, (**c**) x displacements of iFEM-r, (**d**) y displacements of FEM, (**e**) y displacements of iFEM, (**f**) y displacements of iFEM-r.

Figure 14 is a simple diagrammatic sketch for Case 3, and it is a further test based on Case 2. The distributed force is replaced by a single nodal force ($100\sqrt{2}$ MN) at the top corner of the plate. The other parameters and conditions including the sensor locations remain unchanged. The results of the displacements are listed in Table 7. For the full sensor condition, all the percentages are smaller than 1%. For the reduced sensor case, the results of x displacements are still within the 1% range of the FEM results. The y displacements grow slowly to around 4%, which is still accurate. Moreover, the contour plots (Figure 15) of the inverse analysis are identical to the plots of the FEM analysis, which indicates that iQP8 can provide accurate results.

### 3.4. Case 4: Square Plate with a Central Hole under Tension Loading

**Figure 16:**
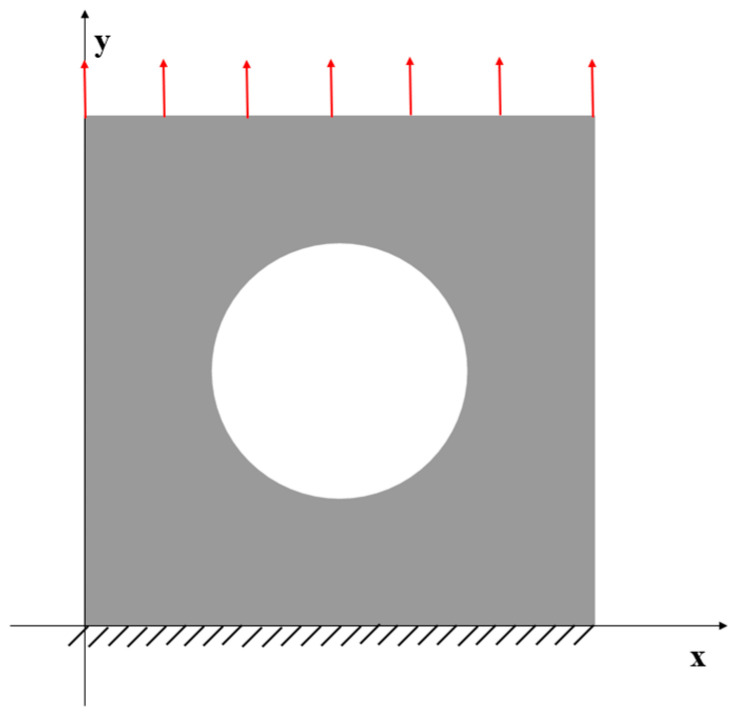
The loading and displacement boundary conditions of Case 4.

**Figure 17:**
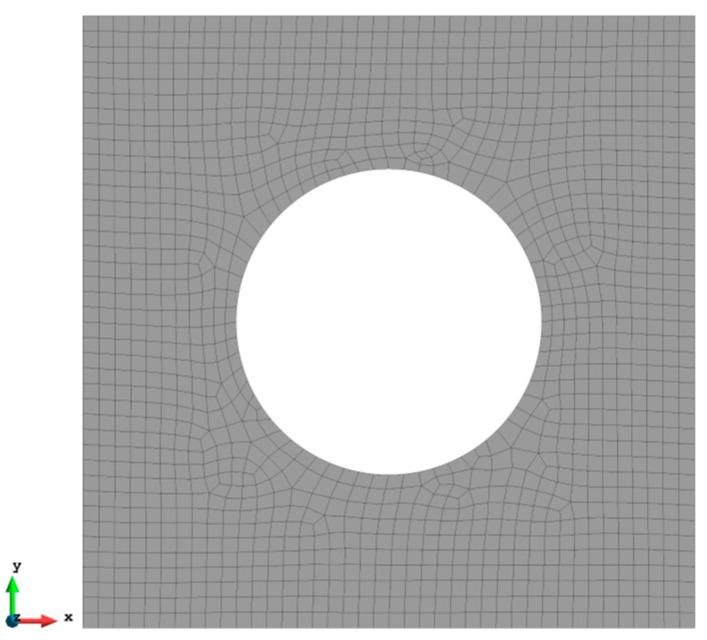
The mesh for Case 4 (1293 elements).

**Figure 18:**
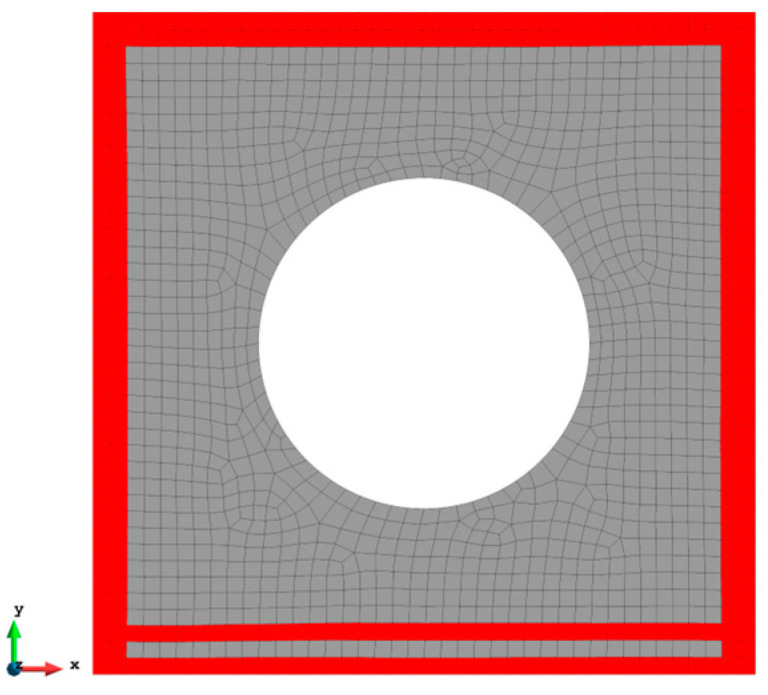
The reduced sensor locations for Case 4 with 304 elements (iFEM-r).

**Figure 19:**
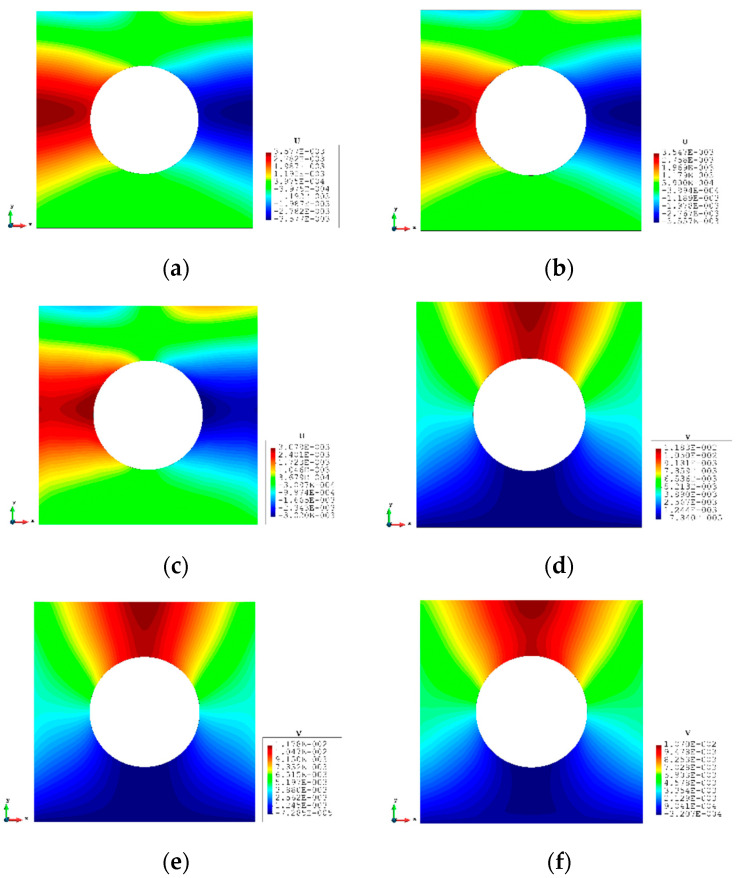
The plots of displacements of Case 4: (**a**) x displacements of FEM, (**b**) x displacements of iFEM, (**c**) x displacements of iFEM-r, (**d**) y displacements of FEM, (**e**) y displacements of iFEM, (**f**) y displacements of iFEM-r.

**Figure 20:**
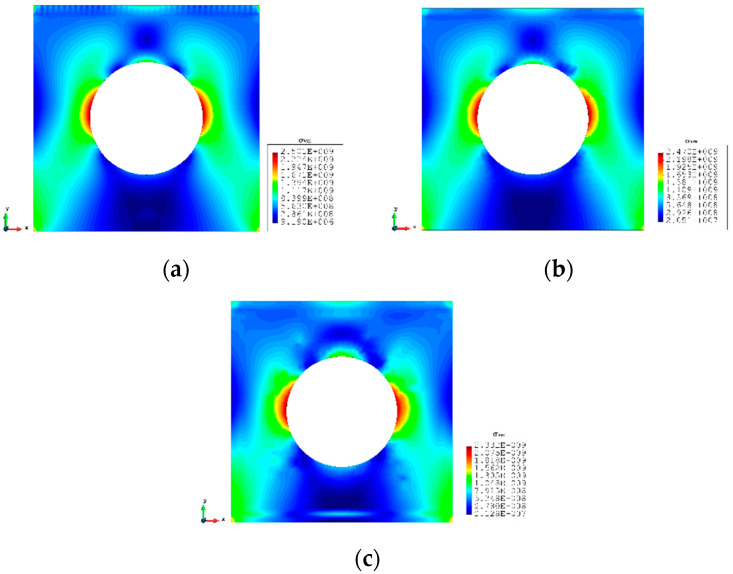
The plots of von Mises stress of Case 4: (**a**) FEM, (**b**) iFEM, (**c**) iFEM-r.

A more complex case which is a plate with a hole at the centre is selected as the last case (Figure 16). The plate has the same geometry as Case 1 and the radius of the hole is 0.5 m. Only fine mesh is considered for Case 4 to ensure the accuracy of FEM analysis, and the plate has been meshed with 1293 elements (Figure 17). The reason for this difference is that around the hole, the mesh would be slightly different, but it will not influence the results. von Mises stress is also chosen for this case to further illustrate the comparison. As shown in Table 8, for the full sensor condition, all three results (u, v, and $\sigma_{vm}$) for both elements are close to the reference FEM results. For instance, the von Mises stress is 1.240% less than the FEM value for the iQP8 element. Moreover, the number of sensors is reduced to 304 as shown in Figure 18. The current sensor locations can provide a less than 10% error for major displacements and von Mises stress. The relatively large percentages of the x displacements can also be explained by the explanation given in Case 2. For the contour plots of Case 4 (Figure 19 and Figure 20), first of all, the plots of the full sensor condition are almost the same as the FEM plots. The main features and tendencies of the plots are captured by the reduced sensor conditions. For example, from Figure 20a,c, it can be seen that the stress is concentrated around the left and right sides of the central hole, and the minimum stress is located around the bottom of the plate. These characteristics are also presented by the FEM plots. The comparison of the results and figures can prove that the iQP8 element even with a limited number of sensors can still estimate accurate results.

## 4. Discussion

In this study, a two-dimensional eight-node quadrilateral inverse finite element formulation, iQP8, is presented. To validate the accuracy of the inverse element and demonstrate its capability, four different numerical cases are considered for different loading and boundary conditions. iFEM analysis results are compared with regular finite element analysis results as the reference solution. For all cases, it was demonstrated that the iQP8 element can provide accurate results even by considering a reduced number of sensors. Therefore, it can be concluded that iFEM and the iQP8 element can be utilised for shape sensing and structural health monitoring of structures under in-plane loading conditions. The presented approached is not only limited to isotropic materials but also can be adapted for monitoring composite materials. For practical applications, the number of sensors can be further reduced as long as the iFEM system can provide sufficient accuracy. The proposed plane element has a computational advantage with respect to the shell element because each node has two degrees of freedom with respect to six degrees of freedom for a shell element if the dominant loading and deformations occur on a particular plane. Moreover, only one sensor is sufficient for each plane element with respect to two sensors (at the top and bottom surfaces) for each shell element.

## Figures and Tables

**Table 1:** Description of numerical cases.

Case 1	Square plate under tension with different mesh
Case 2	Rectangular plate under tension with different mesh
Case 3	Rectangular plate with nodal force and dense mesh
Case 4	Square plate with a central hole and dense mesh

**Table 2:** The results for Case 1 with 16 elements.

Case 1 with 16 elements		Results
*u*	a. FEM	2.267 × 10^−3^
	b. iFEM	2.170 × 10^−3^
Differences between a and b		4.279%
*ν*	c. FEM	2.267 × 10^−3^
	d. iFEM	2.170 × 10^−3^
Differences between c and d		4.279%

**Table 3:** The results for Case 1 with 100 elements.

Case 1 with 100 elements		Results
*u*	a. FEM	2.006 × 10^−3^
	b. iFEM	1.962 × 10^−3^
Differences between a and b		2.193%
*ν*	c. FEM	2.006 × 10^−3^
	d. iFEM	1.962 × 10^−3^
Differences between c and d		2.193%

**Table 4:** The results for Case 1 with 1600 elements.

Case 1 with 1600 elements		Results
*u*	a. FEM	1.786 × 10^−3^
	b. iFEM	1.775 × 10^−3^
	c. iFEM-r	1.772 × 10^−3^
Differences between a and b		0.616%
Differences between a and c		0.784%
*ν*	d. FEM	1.786 × 10^−3^
	e. iFEM	1.775 × 10^−3^
	f. iFEM-r	1.772 × 10^−3^
Differences between d and e		0.616%
Differences between d and f		0.784%

**Table 5:** The results for Case 2 with 125 elements.

Case 2 with 125 elements		Results
*u*	a. FEM	2.539 × 10^−2^
	b. iFEM	2.482 × 10^−2^
Differences between a and b		2.245%
*ν*	c. FEM	1.842 × 10^−3^
	d. iFEM	1.355 × 10^−3^
Differences between c and d		26.439%

**Table 6:** The results for Case 2 with 2000 elements.

Case 2 with 2000 elements		Results
*u*	a. FEM	2.437 × 10^−2^
	b. iFEM	2.421 × 10^−2^
	c. iFEM-r	2.403 × 10^−2^
Differences between a and b		0.657%
Differences between a and c		1.395%
*ν*	d. FEM	1.129 × 10^−3^
	e. iFEM	9.982 × 10^−4^
	f. iFEM-r	1.034 × 10^−3^
Differences between d and e		11.585%
Differences between d and f		8.415%

**Table 7:** The results of Case 3.

Case 3		Results
*u*	a. FEM	3.112 × 10^−2^
	b. iFEM	3.105 × 10^−2^
	c. iFEM-r	3.094 × 10^−2^
Differences between a and b		0.225%
Differences between a and c		0.578%
*ν*	d. FEM	2.106 × 10^−1^
	e. iFEM	2.098 × 10^−1^
	f. iFEM-r	2.026 × 10^−1^
Differences between d and e		0.380%
Differences between d and f		3.799%

**Table 8:** The results of Case 4.

Case 4		Results
*u*	a. FEM	3.112 × 10^−3^
	b. iFEM	3.105 × 10^−3^
	c. iFEM-r	3.094 × 10^−3^
Differences between a and b		0.839%
Differences between a and c		13.922%
*ν*	d. FEM	1.183 × 10^−2^
	e. iFEM	1.178 × 10^−2^
	f. iFEM-r	1.070 × 10^−2^
Differences between d and e		0.423%
Differences between d and f		9.552%
*σ_vm_*	g. FEM	2.501 × 10^9^
	h. iFEM	2.470 × 10^9^
	i. iFEM-r	2.332 × 10^9^
Differences between g and h		1.240%
Differences between g and i		6.757%

## Data Availability

The data presented in this study are available on request from the corresponding author.
